# Improving treatment of people with gastro-esophageal reflux disease refractory to proton pump inhibitors

**DOI:** 10.1038/s43856-024-00632-6

**Published:** 2024-10-14

**Authors:** Joachim Labenz, Sebastian F. Schoppmann

**Affiliations:** 1 Refluxzentrum Siegerland, Siegen, Germany; 2 Medical Faculty of Duisburg-Essen University, Essen, Germany; 3 Department of Surgery, Medical University of Vienna, Vienna, Austria

**Keywords:** Gastro-oesophageal reflux disease, Gastro-oesophageal reflux disease

## Abstract

Proton pump inhibitors (PPIs) are the main treatment recommended and used for gastro-esophageal reflux disease (GERD). However, they fail to control symptoms in a substantial proportion of patients who have PPI-refractory GERD, which is defined as persistent symptoms attributable to objective findings of gastro-esophageal reflux. There remains a lack of dedicated guidelines to direct the management of these patients, some of whom could benefit greatly from surgical treatment. Too often patients remain long-term on ineffective treatment or stop treatment with lack of active review often resulting in their dissatisfaction going unnoticed. Also, concerns over efficacy and side effects of surgical procedures can be off-putting for both patients and physicians. It has been suggested that response to PPIs is predictive of surgical outcome. In this Perspective article we instead recommend that the key determinant should be whether symptoms are caused by GERD. We also discuss the traditional and newer surgical treatment options for people with PPI-refractory GERD.

## Abstract (web summary)

Labenz and Schoppmann discuss the approach to treatment for patients with gastro-esophageal reflux disease that is resistant to standard medical treatment with proton pump inhibitors. They highlight the scope of the problem and the principles of various treatment options with a focus on surgical options, in appropriate patients.

## Introduction

Gastro-esophageal reflux disease (GERD) is the presence of symptoms or complications caused by the backward flow of stomach contents into the esophagus^1^. The pathophysiology is complex and multifactorial and the extent of contribution of each factor remains the subject of discussion among experts^2,3^. In normal physiology, retrograde flow is limited by the anti-reflux barrier, a collection of anatomical and physiological components that act to retain gastric contents. Key components of this barrier are the lower esophageal sphincter, smooth muscle fibers creating a high-pressure zone at the border between the esophagus and stomach; the muscular crural diaphragm that surrounds the diaphragmatic hiatus; and the gastro-esophageal flap valve^2,3^, which is a protrusion of tissue at the lesser curvature of the stomach^4^. Failure of one or all of these components can result in failure of the anti-reflux barrier, leading to increased exposure of the esophagus to gastric juice, containing acid, bile, and enzymes^2^, all of which can induce inflammation and damage^2^. Acid is the key contributor to GERD, with the negative impact being a consequence of increased exposure to stomach acid or increased sensitivity, with the latter resulting in symptoms of GERD in response to a normal amount of acid exposure^2^.

The symptoms of GERD can be highly disruptive to daily life, particularly if they result in sleep disturbance. Complications include esophageal erosions and strictures, and in a small proportion of cases GERD may progress to the premalignant Barrett’s esophagus, a risk factor for esophageal adenocarcinoma.

**Fig. 1:**
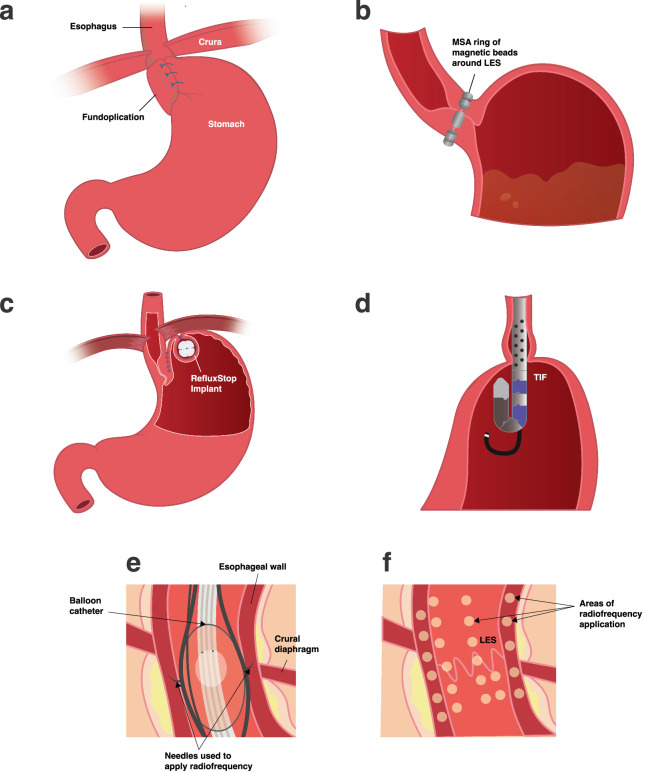
Simplified illustrations of laparoscopic and endoscopic alternatives to traditional fundoplication. **a** Complete (Nissen) Fundoplication, in which the gastric fundus is wrapped around the distal esophagus. **b** Magnetic sphincter augmentation: a magnetic ring of beads placed laparoscopically enhances the lower esophageal sphincter. **c** RefluxStop device is sutured into a pouch on the outside of the stomach fundus, and an esophagogastric plication (sutures) is performed as part of the same procedure, overall keeping the lower esophageal sphincter in an intra-abdominal position. **d** Transoral Incisionless fundoplication, in which the stomach is folded and stapled to the distal esophagus, up to 300 degrees in circumference, to mechanically repair the gastro-esophageal valve. **e**, **f** Stretta system, in which a balloon catheter is inserted into the esophagus. Inflation of the balloon pushes the needles into the esophageal wall, where radiofrequency thermal energy is delivered to multiple locations near the gastro-esophageal junction to induce tissue hypertrophy and thus improve the acid barrier.

In broad terms, the main aims of treatment have been to pharmacologically reduce the acidity of refluxate or to surgically repair the failed anti-reflux barrier through hiatal hernia repair and fundoplication, in which the gastric fundus is wrapped around the distal esophagus to reinforce the lower esophageal sphincter and to keep the stomach below the diaphragm (Fig. 1a). The introduction of proton pump inhibitors (PPIs) which act to block the final common pathway of secretion of gastric acid from parietal cells resulted in substantial progress in the treatment of GERD. These medications offered several advantages over the previously available treatments, including potency, effect duration, improved postprandial and nocturnal control, and lack of tachyphylaxis (a reduction in effectiveness with repeated exposure)^5^.

For many patients, PPIs allow effective symptom control and healing of inflammation of the esophagus (esophagitis). Whilst their introduction, over 30 years ago, was very successful, their limitations have become increasingly recognized in recent years. It was initially thought that long-term PPI therapy could be an effective and appropriate treatment for chronic GERD^6^, including the suggestion that a diagnosis of GERD should be based on response to PPIs^6,7^. More recently potential side effects with long-term use have been highlighted, and, importantly, it is reported that PPIs fail to control symptoms in 30–40% of patients^8–11^.

Here we discuss the impact of this treatment-resistant disease and suggest management options to optimize the care of this group of patients. We discuss pertinent research findings that may influence treatment decisions and highlight the potential role of surgery, an option that is often overlooked.

## Impact of GERD

Persistent GERD symptoms are associated with anxiety, decreased quality of life, and decreased ability to work. It is estimated that the prevalence of GERD is 8.8–25.9% in Europe and 18.1–27.8% in North America^12^. Thus, ineffective treatment has wide-reaching implications for treatment cost, with increasing disease severity increasing costs and work absence^13–15^. An analysis based on the German system, which assumed a 15% prevalence rate, suggested around 600 million euros loss in gross domestic product was attributable to GERD under routine clinical care, increasing to more than 1 billion euros when also including the impact of untreated GERD^16^.

Determining the exact prevalence of PPI failure is challenging since definitions of persistent symptoms vary. In addition, some patients give up attempting to get treatment and do not pursue further medical assistance. Controlled studies with PPIs frequently report heartburn as the primary outcome measure. Since heartburn is much more likely to respond to PPIs compared to regurgitation, it is likely that the success of PPI treatment is overestimated. In a systematic review of studies set in primary care (mainly European countries and the USA)^17^, rates of partial response or nonresponse to PPIs (defined as troublesome heartburn or regurgitation) ranged from 17 to 32% in interventional studies but were as high as 45% in observational studies^17^. In a USA population-based study from 2020, of the 3229 participants taking daily PPIs, 54.1% had persistent GERD symptoms^18^.

In addition, any status reported by physicians rather than patients themselves is likely to underestimate symptoms. El Serag et al. ^17^ noted that the lowest reported rates of partial- or nonresponse (8% and 12%) came from two studies^19,20^ in which symptoms were assessed by physicians as opposed to patients. We previously found that one-fifth of patients were very dissatisfied with their current long-term PPI treatment and about 50% complained of persistent symptoms including sleep problems at least twice weekly, yet this often went unrecognized by their primary care physician^21^. A key message from this study^21^ was the importance of actively reviewing patients to ask them about symptom control. Patients in the primary care setting, which is where most patients are managed, are often issued a repeat prescription over the phone without critical consideration of whether the medication should be continued. This occurs even when these patients may benefit from a different or optimized medical treatment or surgery. In reality, only a minority reach the stage of surgical treatment. Low levels of further investigation and referral for surgical assessment have been found which result in less than 0.1% of people in Germany with GERD (>10 million annually) undergoing surgery^21^. A review paper also highlighted that despite aggressive acid suppression therapy failing to control symptoms in approximately 40% of GERD patients, less than 5% undergo fundoplication surgery^22^.

## Surgical assessment

There seems to be hesitancy to refer patients for surgical assessment. Hesitancy is probably due to uncertainty about which patients to refer, a view that it will not be effective, and concern regarding side effects. A 2016 review of surgical options acknowledged a therapy gap^23^ caused by the combination of PPI-refractory symptoms and patients’ reluctance to undergo surgery for fear of side effects. Regarding the selection of patients to refer, Thompson and Watson^24^ added to existing recommendations^25^ by suggesting surgical referral criteria also include patients with predominantly volume reflux, patients with continued endoscopic findings of esophagitis despite maximal medical therapy, and patients with mechanical issues such as symptomatic large hernia^24^. These criteria are in addition to existing recommendations^25^ for patients with PPI intolerance and/or legitimate concern regarding PPI side effects, those with proven evidence of reflux disease not wishing to take PPIs, and those with breakthrough or nonresponsive symptoms despite maximal medical therapy^24,25^. We agree entirely with their recommendations. Furthermore, symptom index (the percentage of symptom events preceded by reflux episodes on pH monitoring)^26,27^ and symptom association probability (a statistical method for the correlation of symptoms with reflux events)^28^ should be clarified to establish whether remaining symptoms are linked to reflux events^6,29^. This enables increased prediction of the success of anti-reflux interventions.

In addition, there is a perception that a good response to PPIs is required for a successful surgical outcome. In 1999 symptomatic relief following laparoscopic Nissen fundoplication was analyzed for predictors of surgical success using data from 233 patients^30^. The analysis found that the worst chance of successful outcome (odds ratio [OR], 1.0) was conferred with the combination of a normal 24-h pH score, atypical primary symptoms, and poor or no response to acid suppression therapy, while the best chance of good outcome occurred with the combination of abnormal 24-h pH score, typical symptoms (heartburn, regurgitation and dysphagia), and complete or partial response to acid suppression therapy (OR, 89.0). It is worth noting, however, that poor or no response to acid suppression therapy alone did not equate to an unsuccessful outcome. For example, the combination of typical symptoms and abnormal 24-h pH score still gave an OR of 27.2 for a successful outcome. On rank ordering the factors predictive of outcome, abnormal 24-h pH monitoring score was the most powerful (OR, 5.4), followed by typical primary symptoms (OR, 5.1), then complete or partial response to acid suppression therapy (OR, 3.3)^30^.

Ten years later, a systematic review of predictors of clinical outcomes following fundoplication which included 53 cohorts and 10 case-control studies found no consistent associations between preoperative response to acid suppression medications, baseline symptoms, baseline acid exposure, degree of lower esophageal sphincter competence, or position of reflux and surgical outcomes^31^. The need for better study design was also highlighted because previously described associations were based on mixed quality and consistency of data^31^.

More recent study findings appear to contradict the idea that PPI resistance is predictive of surgical failure, finding surgical treatment to be superior to medical treatment in those who had truly PPI-refractory GERD^32^. The study analyzed patients who had reflux-related PPI-unresponsive heartburn, defined as no response to 2 weeks of 20 mg omeprazole twice daily followed by an investigation to confirm reflux. Patients were randomized to receive surgical or one of two medical treatments. Medical treatment was deemed active, comprising omeprazole 20 mg bid plus baclofen ± desipramine, or control, comprising omeprazole 20 mg (bid plus placebo). The first key finding was that few of the patients (39%) actually had PPI-refractory heartburn caused by reflux, with the other diagnoses including non-GERD disorders, such as eosinophilic esophagitis, and functional heartburn. Then, among those who were randomized, surgery gave better outcomes (67% treatment success versus 28% for the medical group, *P* = 0.007)^32^. An important point from this study is that the patients deemed to have had successful outcomes had true PPI-refractory GERD. This study highlights the importance of ensuring patients undergo further assessment and, if appropriate, consideration for surgical treatment, rather than only having access to a medical treatment that does not work for them.

Very recently, an observational study of 73 patients found that partial response to PPIs, compared to no response, was a protective factor against dissatisfaction with laparoscopic anti-reflux surgery outcome^33^. Nonetheless, in our opinion, the idea that a lack of response to PPIs means surgery will be ineffective, or that only patients who respond to PPIs are candidates for surgery, is potentially damaging, since this group of patients forms a core demographic that could benefit from additional treatment options, provided they have objectively demonstrated GERD.

Previous guidelines have generally neglected to address the management of patients with PPI-refractory GERD, as acknowledged in ref. ^34^. One of us (J.L.) was recently involved in developing guidelines for the German Society for Gastroenterology, Digestive and Metabolic Diseases (DGVS)^35^. Others have also highlighted the existence of this patient group^9,11,36,37^ and generally agree on the need for treatment targeted to the underlying mechanism, however, they have often focused on optimizing medical management. We think that there is still a lack of awareness and potential underutilization of surgical options. In light of this, we recommend the following investigative workup of these patients.

## Proposed investigations and treatment options

**Fig. 2:**
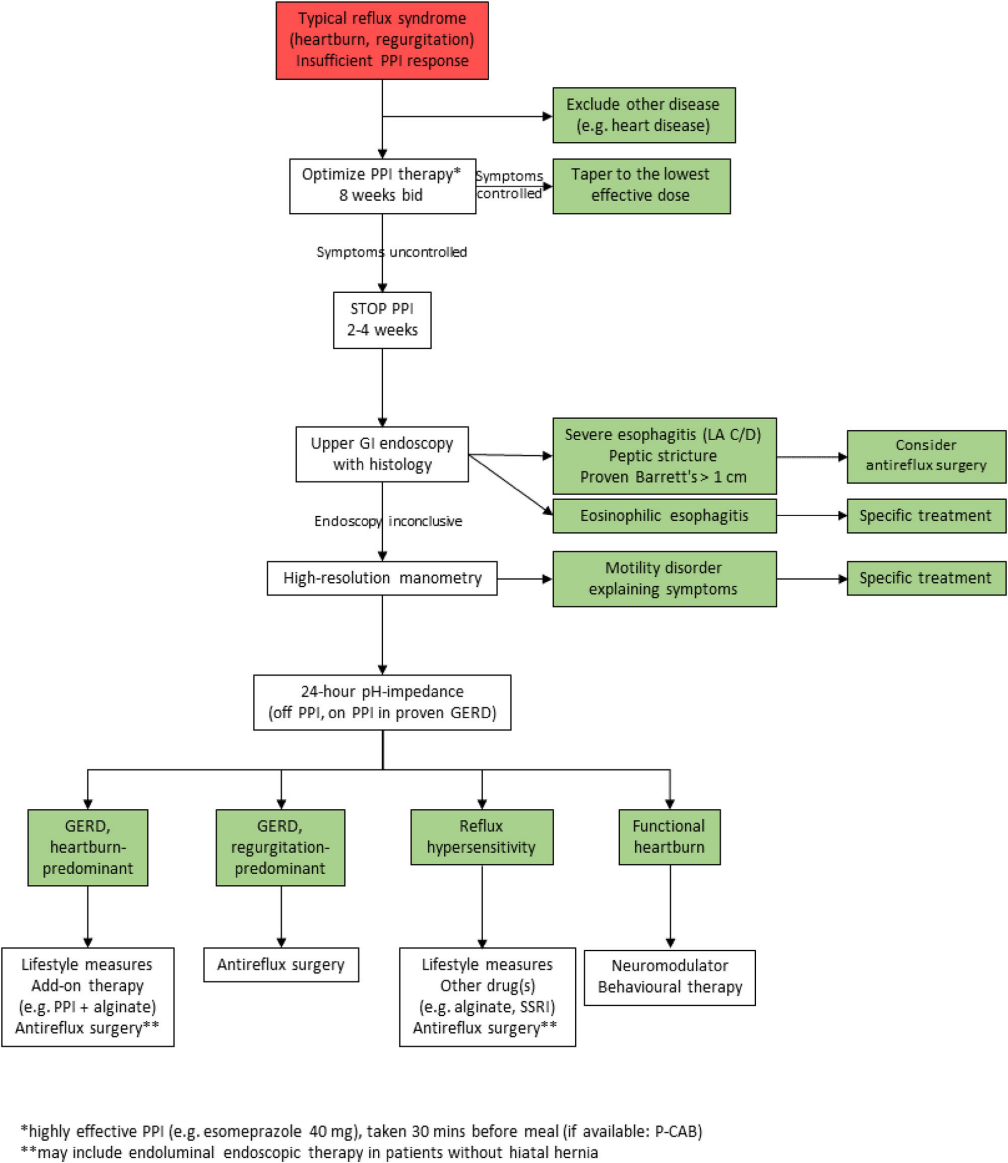
Flowchart of recommended investigations and treatment for patients with persistent symptoms suggestive of GERD with insufficient response to PPIs. Patients who present with typical reflux symptoms should have an 8-week trial of PPI therapy. If ineffective, PPI should be stopped and stepwise investigations performed: upper GI endoscopy followed by high-resolution manometry and 24-h pH-impedance testing, with treatment according to investigation results. bid bis in dia (twice daily), GERD gastro-esophageal reflux disease, GI gastrointestinal, LA C/D Los Angeles classification system grade C/D, P-CAB potassium-competitive acid blockers, PPI proton pump inhibitor, SSRI selective serotonin reuptake inhibitor.

There are multiple possible causes of heartburn that persist despite PPI treatment^32^, including non-GERD-related causes, treatment compliance issues, residual acid reflux, weakly acidic and weakly alkaline reflux, and functional heartburn^37^. Firstly, and of utmost importance, two crucial points must be confirmed: that the patient actually has GERD, confirmed by objective testing; and that it is refractory to PPIs. Spechler et al. found only 78 of the 366 patients enrolled in their study had true PPI-refractory GERD^32^ with the remainder having causes of symptoms other than GERD or that showed improvement with a trial of omeprazole^32^. Figure 2 shows the steps that we recommend be followed when assessing patients with symptoms suggestive of GERD.

Other more likely diagnoses should be ruled out, including red flag symptoms, that could be indicative of another serious health issue. Assuming the patient has had 8 weeks of standard-dose PPI, it is sensible to try an 8-week period of twice-daily dosing or change to another more potent PPI such as esomeprazole. These must be taken correctly, 30–60 min before breakfast and evening meal^36^.

The management of patients whose symptoms respond to PPIs is dealt with in other articles and guidelines^25,38^; we focus here on refractory GERD only. If symptoms persist, then further investigation is warranted. Endoscopy and possibly biopsy should be undertaken in patients who have not taken PPIs for 3–4 weeks to look for Barrett’s esophagus, eosinophilic esophagitis, and grade C/D esophagitis^35^. If none of these are found, high-resolution manometry should be undertaken to rule out motility disorders. If no relevant motility disorder is found, PPI can be resumed in patients with previously proven GERD, and 24-h pH-impedance should be carried out. Normal 24-h impedance indicates potential diagnoses of esophageal hypersensitivity or functional heartburn. Briefly, esophageal hypersensitivity may be treated with lifestyle modification, alginate, baclofen, selective serotonin reuptake inhibitor (SSRI), or anti-reflux surgery in selected cases, while functional heartburn may be treated with neuromodulator or behavioral therapy.

However, the focus of this article is patients who have GERD and PPI resistance. Therefore, if pathological acid reflux is found, options are general measures to optimize acid inhibition, such as adding another type of therapy, for example, an alginate, or considering anti-reflux surgery. It should be borne in mind that PPIs work to change the content of refluxate but do not reduce reflux volume or frequency. In contrast, surgical options have this as their aim and thus constitute a causal treatment rather than the amelioration of one relevant pathogenic factor in the complex pathophysiology of GERD.

Based on our considerations and the evidence, we advocate for a move away from using response to PPI as a criterion for a patient’s suitability for surgery. Rather, the main criterion for surgery should be proven GERD, in which reflux is objectively identified with suitable investigations as the cause of the symptoms. This is compatible with prior observations that the amount of certainty that gastro-esophageal reflux is the underlying cause of a patient’s complaints predicts whether laparoscopic fundoplication is a successful treatment^30^.

The side effects of surgery should be recognized, bearing in mind that they are generally short-lived and manageable. There are now several different laparoscopic and endoscopic options. Laparoscopic fundoplication (Fig. 1a) is a well-established standard of care in many countries, and one of its strengths is that long-term data are available^39^. The 2021 SAGES (Society of American Gastrointestinal and Endoscopic Surgeons) guidelines on the surgical treatment of gastro-esophageal reflux suggest that adult patients with confirmed chronic or chronic refractory gastro-esophageal reflux should be managed with surgical fundoplication rather than continued medical treatment^34^. Note that this is a suggestion rather than a firm recommendation due to the quality of evidence being low. It derives from the expert panel judging that the moderate desirable effects of surgery over medical management outweigh the small undesirable effects, based on literature on percentage time with abnormal pH, PPI usage, short-term quality of life, and long-term symptom control. Within this guideline^34^ the lack of detail provided in earlier guidelines on surgical treatment options is acknowledged. The guideline considers different types of surgery, including partial versus complete fundoplication, as well as technical aspects of fundoplication, such as robotic versus laparoscopic and division of the short gastric vessels. A debate of the superiority of particular surgical approaches and steps or versions, such as total versus partial fundoplication, is beyond the scope of this paper given this issue is also assessed in other guidelines such as 2022 UEG (United European Gastroenterology) and EAES (European Association for Endoscopic Surgery) rapid guideline on surgical management of GERD^40^. In general, there have been multiple studies comparing the different extents of fundoplication^39,41^, and ultimately this decision must be made by the surgeon and patient. However, we wish to highlight that this area continues to spark discussion, particularly regarding the best option when there is concern about dysphagia.

Besides laparoscopic fundoplication in its various forms, those responsible for the care of GERD patients should ideally have an awareness of other surgical and endoscopic options as these may alleviate some of the apprehension around surgery. Generally, these techniques have been developed in an attempt to address some of the shortfalls of fundoplication, such as side effects of dysphagia, bloating, inability to belch or vomit, and those related to surgical frailty. Unfortunately, there are few head-to-head studies comparing these newer procedures. In addition, regional differences in practice are likely to exist, including differences in funding and payment practices, which may be a barrier if systems will not pay for a particular procedure.

Amongst these alternatives to fundoplication (Fig. 1a) are the laparoscopic surgical procedures of magnetic sphincter augmentation (MSA) (Fig. 1b), the RefluxStop implant (Fig. 1c), and EndoStim pacing, and the endoscopic procedures of transoral incisionless fundoplication (TIF) (Fig. 1d), GERD-X, anti-reflux mucosectomy (ARMS), and the Stretta procedure (Fig. 1e, f). Importantly, the endoscopic options are only suitable for patients without hiatal hernia. The key points of these individual procedures are detailed below, with a view to providing a basic understanding of the concepts behind them and key evidence or lack thereof. It should be noted that some of these are still considered experimental, plus vary in their availability according to geographical region.

## Surgical alternatives

In MSA (also known as the Linx Reflux Management System), a flexible ring of magnets is placed around the circumference of the esophagus (Fig. 1b). The technique has evolved somewhat and may now include a formal crural repair^42^. A previous review reported that its efficacy was approaching that of Nissen, with fewer side effects, though noted that studies were limited to a select population^23^. A 2017 SAGES panel analysis concluded that it gave similar rates of success in treating GERD symptoms as laparoscopic fundoplication, possibly with lower side effects of bloating^43^. The 2022 UED and EAES guideline^40^ only included randomized controlled trials but did not include controlled trials of MSA as the follow-up was too short (12 months). In said 12-month study, Bell et al. compared outcomes with MSA versus PPIs and found MSA to be superior, with results sustained at 1 year^44^, but the study was biased by including only patients with regurgitation not responding to a PPI. Ferrari et al. have published 6- to 12-year outcomes from 124 patients (median follow-up 9 years) from a prospective single-center registry. They reported that 89% of patients had a favorable long-term outcome (based on GERD-HRQL reduction) and 92.7% reported satisfaction^42^. The device is contraindicated in patients with electrical implants such as defibrillators or pacemakers or metallic implants in the abdomen^43^.

The RefluxStop procedure involves the implantation of a nonactive device, roughly 25 mm, on the outer surface of the stomach, to keep the lower esophageal sphincter within the abdominal cavity, while allowing a degree of movement with the diaphragmatic movements of breathing (Fig. 1c). One of the main theoretical attractions of this procedure is that it was designed to avoid encircling or compressing the esophagus, with a view to limiting the risk of dysphagia. The procedure itself includes hernia repair as standard. Currently, data up to 4 years have been published, which showed a median reduction in GERD-HRQL score (GERD health-related quality of life, a measure of symptom burden) of 90% from baseline and 95.5% (42/44 patients) reporting satisfaction^45^. Longer-term outcomes with more patients are required. The device is CE-marked but not currently FDA-approved.

EndoStim is a method that is not currently available in which the lower esophageal sphincter is stimulated using electricity to increase the sphincter tone^46^. In patients with a hernia, appropriate repair is recommended. Since this procedure involves only narrowing the hiatus with one or two stitches, the long-term stability of the repair is uncertain. Patients have to be monitored over time with adjustment of the electric stimulation and repeat operations, for example at the end of the life of the stimulating device. The method has demonstrated efficacy and reasonable safety in uncontrolled small-scale studies^46,47^ but data from a randomized, sham-controlled study conducted according to FDA recommendations is not available^47^. A careful approach with regard to patient selection and education, surgical training, and conduct in expert centers is warranted^48^. It is contraindicated for those with significant cardiac arrhythmia or ectopy or cardiovascular disease, and multiple sources are listed as potentially interfering with the device^49^.

## Endoscopic alternatives

TIF involves neo-valve reconstruction^50^ by folding and stapling the stomach to the distal esophagus, up to 300 degrees in circumference^50^, in an attempt to mechanically repair the gastro-esophageal valve (Fig. 1d)^51^. A recent systematic review and meta-analysis reported pooled patient satisfaction of 70.6%, and a significant and persistent improvement in symptoms and quality of life in four-fifths and two-thirds of patients at 4–5 and 10 years, respectively, concluding that it is a safe option for those who cannot have or do not want to have long-term medical therapy or surgery^52^. The technique is less invasive and does not require postoperative dietary restrictions. Laparoscopic hernia repair may or may not be performed alongside the procedure depending on the patient’s anatomy.

GERD-X is an endoscopic full-thickness fundoplication (EFTP) device^53^. In EFTP, transmural sutures at the gastro-esophageal junction are used to restructure the gastric cardia anatomy and strengthen the valve mechanism. GERD-X, available since 2014, was developed with the aim of simplifying the procedure. A sham-controlled trial published in 2022 demonstrated its safety and efficacy^53^.

The Stretta system delivers radiofrequency thermal energy to several locations close to the gastro-esophageal junction, to induce tissue hypertrophy, and thus improve the acid barrier (Fig. 1e, f)^54^. A lack of consistent and long-term outcome data means that bodies such as the American College of Gastroenterology (ACG)^38^ and the National Institute for Clinical Excellence are reluctant to recommend it other than by special arrangement or as part of research^55^.

A standardized technique is not yet available for endoscopic mucosa resection of the cardia or anti-reflux mucosectomy (ARMS)^54^. The underlying mechanism is poorly understood^56^ but it has been postulated that it creates a degree of stricture at the gastric cardia^57^. It is more appropriate for those who do not have a significant sliding hiatal hernia^56,57^.

## Call to action

Overall, the choice of whether or not to undergo surgery and the type of surgery that could offer the most benefit to an individual patient should be decided between the surgeon and the patient, taking into account not only the surgeon’s experience but also the likelihood of side effects. We should avoid a blanket approach and seek to determine exactly what abnormalities are present and then consider carefully the treatments that specifically address the underlying abnormalities. Patient preferences and response to prior therapies should also be taken into account^58^ but we must be cautious that they are not misused as a barrier to surgical treatment.

We urge those responsible for the continuing care of these patients, such as primary care physicians and gastroenterologists, to remain aware that these surgical options exist and are constantly evolving, and to consider surgical referral in patients who have persistent symptoms caused by GERD despite correct prescribing of and adherence to PPI therapy. This patient group is not appropriately treated by long-term PPI medication, but they will only be identified if they are regularly and actively reviewed. Scientific societies also have a role to play in including newer surgical alternatives in their evidence guidelines, particularly as evidence continues to grow in terms of follow-up and larger patient groups.

In our view, patients with PPI-refractory reflux symptoms should be managed by interdisciplinary reflux teams that provide general and nutritional advice, specialized workup with the expertise of technicians and gastroenterologists, and input from upper gastrointestinal surgeons with sufficient experience in anti-reflux surgery. It has been shown that the number of anti-reflux procedures per year is crucial with respect to the overall safety profile of fundoplication^59^. We strongly advocate the new endoscopic and surgical interventions to be introduced first in experienced centers. We firmly believe that a better understanding of the pathophysiology of the disease and the identification of reason(s) for PPI failure will allow a tailored interventional therapy with a high rate of success and low risk for the patients.
